# Wetland restoration suppresses microbial carbon metabolism by altering keystone species interactions

**DOI:** 10.3389/fmicb.2025.1570703

**Published:** 2025-04-30

**Authors:** Huijie Zheng, Deyan Liu, Ye Li, Zengming Chen, Junjie Li, Yanhong Dong, Cong Yang, Yuncai Miao, Junji Yuan, Weixin Ding

**Affiliations:** ^1^State Key Laboratory of Soil and Sustainable Agriculture, Institute of Soil Science, Chinese Academy of Sciences, Nanjing, China; ^2^University of Chinese Academy of Sciences, Beijing, China; ^3^Co-Innovation Center for Sustainable Forestry in Southern China, Nanjing Forestry University, Nanjing, China; ^4^University of Chinese Academy of Sciences, Nanjing, China

**Keywords:** agricultural abandonment, bacterial diversity, co-occurrence network, carbon metabolism, soil organic carbon decomposition, wetland restoration

## Abstract

Soil bacteria play a pivotal role in regulating multifaceted functions of terrestrial ecosystems. Unraveling the succession of bacterial communities and the feedback mechanism on soil organic carbon (SOC) dynamics help embed the ecology of microbiome into C cycling model. However, how wetland restoration drives soil bacterial community assembly and species association to regulate microbial C metabolism remains unclear. Here, we investigated soil bacterial diversity, community structure and co-occurrence network, enzyme activities and SOC decomposition in restored wetlands for one, three, and four years from paddy fields in Northeast China. Wetland restoration for three and four years increased taxonomic (richness) and phylogenetic diversities by 2.39–3.96% and 2.13–3.02%, respectively, and increased the relative contribution of nestedness to community dissimilarity, indicating increased richness changed soil bacterial community structure. However, wetland restoration for three and four years decreased the richness index of aerobic Firmicutes by 5.04–5.74% due to stronger anaerobic condition characterized by increased soil Fe^2+^/Fe^3+^ from 0.20 to 0.64. Besides, wetland restoration for four years decreased network complexity (characterized by decreased node number by 2.51%, edge number by 9.62%, positive/negative edge number by 6.37%, average degree by 5.74% and degree centralization by 6.34%). Robustness index decreased with the increase of restoration duration, while vulnerability index increased with the increase of restoration duration, indicating that wetland restoration decreased network stability of soil bacterial communities. These results might be because stronger anaerobic condition induced the decrease of aerobic Bacilli richness index in keystone module, thereby reducing positive association within keystone module. Decreased positive species association within keystone module in turn weakened microbial C metabolism by decreasing hydrolase activities from 7.49 to 5.37 mmol kg SOC^−1^ h^−1^ and oxidase activities from 627 to 411 mmol kg SOC^−1^ h^−1^, leading to the decrease of SOC decomposition rate from 1.39 to 1.08 g C kg SOC^−1^ during wetland restoration. Overall, our results suggested that although wetland restoration after agricultural abandonment increased soil bacterial diversity, it decreased positive association within Bacilli-dominated keystone module under stronger anaerobic condition, which weakened microbial C metabolism and SOC decomposition.

## 1 Introduction

Wetlands hold high biodiversity and soil organic carbon (SOC) sequestration, thereby providing multiple ecosystem services (Mitsch and Gosselink, 2015). In past three centuries, more than 20% of global wetlands have been converted into farmlands (Fluet-Chouinard et al., 2023). Accordingly, the restoration of wetlands worldwide is imperative to utilize ecological functions and values of wetlands (Craft, 2022). However, previous studies showed that wetland restoration increased, decreased and did not change SOC content and storage (Bonetti et al., 2021; Zhang S. et al., 2024), mainly because the influencing factors of SOC mineralization are unclear. Therefore, there is a need to elucidate the mechanism of SOC decomposition in order to further develop effective management strategies for SOC accumulation during wetland restoration.

Extracellular enzymes such as hydrolases and oxidases play important roles in orchestrating SOC decomposition (Luo et al., 2017). During wetland restoration, stronger anaerobic condition was suggested to suppress oxidative enzymes, which in turn favored the accumulation of some phenolic compounds that can be detrimental to the hydrolytic enzymes involved in the decomposition of macromolecules based on “enzyme latch” theory (Freeman et al., 2004; Zak et al., 2019). Not only that, soil enzymes, which are mainly generated by microorganisms, serve as “sensors” for physicochemical conditions, and may establish valuable linkages between microbial communities and C cycling (Trivedi et al., 2016; Neemisha and Sharma, 2022). For example, a meta-analysis showed that afforestation from cropland, grassland, bare land and pasture enriched oligotrophic microorganisms due to the increase of plant C inputs, and induced the increase of soil enzyme activities but the decrease of metabolic quotient (Luo et al., 2023). However, the impact of microbial communities and functional guilds on the decomposition rate and fate of SOC is limited during wetland restoration.

Previous studies revealed that soil microbial diversity and community composition regulated their metabolic capacities (Torsvik and Øvreås, 2002; Liu et al., 2018). De Graaff et al. (2015) observed that loss of microbial diversity strongly reduced SOC decomposition. Besides, salinization in a subtropical tidal wetland induced higher C-degrading enzyme activities by increasing the prevalence of oligotrophs over copiotrophs (Chen et al., 2022). However, Shu et al. (2023) suggested that increased SOC mineralization was related with the relative abundances of Proteobacteria and Bacteroidetes under ecological restoration. Therefore, it is argued that depending exclusively on the assessment of soil microbial diversity or community composition to comprehend SOC decomposition was far from sufficient (Wang et al., 2021; Wang Q. et al., 2023).

Species association has been proposed as an important complement to ecological functionality of soil microbial communities (Gaüzère et al., 2022), as they influenced soil microbial community structure through synergistic and competitive interactions to achieve efficient resource utilization and enhance resilience advantage (Zelezniak et al., 2015; Maynard et al., 2017). Li et al. (2024) observed that agricultural cultivation strengthened competitive and predator–prey interactions, modulated their microspatial distribution, and increased connection with organic resources that in turn accelerated SOC decomposition. Ecological clusters of co-occurrence networks (also known as modules) are substructures of functional guilds performing specific metabolic activities synergistically (Dundore-Arias et al., 2023). Shi et al. (2024) found that the module dominated by metabolic interactions between Sphingomonas and associated species was related closely to the decomposition of SOC especially recalcitrant organic C compounds during long-term vegetation succession. Therefore, species associations of modules play vital roles in soil C cycle and must be taken into account during wetland restoration.

Soil physicochemical properties affected SOC decomposition by changing microbial communities and enzyme activities (Jing et al., 2017; Zhang et al., 2021). For example, Firmicutes, Actinobacteria and Proteobacteria dominated SOC decomposition after plant straw inputs (Xiao et al., 2022). Zhang Y. et al. (2024) found that the main factors affecting SOC mineralization under drought stress were phosphatase activity and Chloroflexi relative abundance, while the main factor affecting SOC mineralization under water stress was Ascomycota relative abundance. The relative abundance of Gemmatimonadetes was the main limiting factor of SOC mineralization in wet and dry cycles. Therefore, understanding the relationships between soil physicochemical properties, microbial communities and enzyme activities could improve the ability to accurately predict SOC decomposition during wetland restoration.

Sanjiang Plain represents the most extensive restored wetland region in Northeast China, accounting for 61% of national restored wetlands (Mao et al., 2018). Our previous results showed that wetland restoration promoted the accumulation of plant-derived C in SOC, partly due to decreased microbial C metabolism under stronger anaerobic condition (Zheng et al., 2024). Here, we measured SOC decomposition and soil bacterial community characteristics in restored wetlands for one, three, and four years from paddy fields. The aims of this study were to (1) understand the variations of soil bacterial diversity, community structure, and co-occurrence network; and (2) elucidate the endogenous regulatory influences of soil bacterial community succession on microbial C metabolism and SOC decomposition. We hypothesized that wetland restoration after agricultural abandonment promoted the growth of anaerobic species, while inhibited the reproduction of aerobic species and their associations of soil bacterial communities that weakened microbial C metabolism and SOC decomposition due to stronger anaerobic condition.

## 2 Materials and methods

### 2.1 Study site and soil sampling

The study area (45°24′9′′N, 132°56′21′′E) is situated in Heilongjiang Province, China. The region is characterized by a cold temperate continental monsoon climate, with a mean above-sea level, annual temperature and precipitation of 54 m, 4.37°C and 667 mm, respectively. In the late Pleistocene, wetlands were formed by the confluence of Heilongjiang River, Songhua River, and Wusulijiang River. In 2013, these wetlands were transformed into paddy fields, which were irrigated in May and drained before harvest in September. Since October 2018, however, the region had implemented a wetland restoration project, building dams to prevent drainage, and allowing the natural regeneration of vegetation. The standing water depth of restored wetlands was 20–40 cm and the vegetation type was *Deyeuxia angustifolia*.

In this area, soils were sampled in paddy fields (RF) and wetlands at one (RW1), three (RW3), and four (RW4) years of restoration from October 2018 to October 2022 (except October 2020). At five sampling plots of each sampling time, the topsoils (0–20 cm) were collected using augers from 10 sampling sites, and carefully mixed. Next, visible plant and gravel residues were removed in the laboratory using forceps. All soils were divided into three subsamples. The first two were air-dried and freshly used for analysis of soil properties. The last sample was freeze-dried to analyze soil microbial community structure.

### 2.2 Determination of soil properties

Soil pH was measured in a suspension with the ratio of soil and water of 1:5 (weight:volume) using a Seven Compact pH meter (Mettler Toledo, Greifensee, Switzerland). Soil ferrous iron (Fe^2+^)/ferric iron (Fe^3+^) was used as the proxy of soil redox condition as in many previous studies (Bai et al., 2021; Marschner, 2021). Dissolved organic C (DOC) was quantified on a total organic C analyzer (Elementar, Hanau, Germany). Soil inorganic nitrogen (N) content was quantified on a San++ System segmented flow analyzer (Skalar Analytical BV, Breda, Netherlands). The contents of SOC and total N (TN) were quantified using a Vario Max CN analyzer (Elementar, Hanau, Germany). Total phosphorus (TP) was extracted through acid digestion, and the content was measured using a UV1800-visible spectrophotometer (Shimadzu, Kyoto, Japan). Detailed methods for contents of soil Fe^2+^, Fe^3+^, DOC and inorganic N were depicted in attachment materials (Supplementary method 1). These soil properties were showed in Supplementary Table S1.

### 2.3 Determination of soil enzyme activities and SOC decomposition

The activities of hydrolases (*β*-1,4-glucosidase and cellobiohydrolase) were measured according to the method of Burns et al. (2013). The activities of oxidases (peroxidase and phenol oxidase) were measured according to the method of DeForest (2009). Detailed methods were depicted in attachment materials (Supplementary method 2). Using the activities of hydrolases and oxidases normalized with SOC content, an integrated index reflecting microbial C metabolism was established based on principal component analysis. This index was characterized by the score of the first axis, which explained 92.1% variance as it was positively related with the activities of hydrolases and oxidases (*p* < 0.05; Supplementary Figure S1).

SOC decomposition was measured using a microcosm incubation experiment (Tang et al., 2024). Briefly, 2.5 g of soil samples (on an oven-dried basis) and 37.5 mL of distilled water were placed in 100 mL airtight jars. The headspace gas was replaced with N_2_, after which the jars were securely sealed with a butyl plug and an aluminum foil cover. The samples were incubated for 25 days at 20°C, which represented average soil temperature from May to September, a timeframe that dominated SOC decomposition in this area (Chen et al., 2017). Gas samples were extracted at day 1, 2, 3, 5, 7, 10, 17, and 25. During each sampling, 20 mL homogenized headspace gas was drawn using syringes. The concentrations of C dioxide and methane were determined using a gas chromatograph (Agilent, California, USA). The SOC decomposition was represented by cumulative gas emission and normalized with SOC content.

### 2.4 High-throughput sequencing

DNA was extracted from 0.5 g freeze-dried soils using FastDNA Spin Kit for Soil (MP Biomedicals, CA, USA). The target gene was amplified using primers 515F/907R (Jiao et al., 2018). Purified amplicons were paired-end sequenced by Illumina MiSeq platform. Fastq files were filtered by Trimmomatic and merged by FLASH. Operational taxonomic units (OTUs) were clustered by Usearch. The classification of each OTU was annotated using the Silva database. Detailed methods were depicted in attachment materials (Supplementary method 3). In addition, the tool “phylogenetic investigation of communities by reconstruction of unobserved states 2 (PICRUSt2)” was used to predict the functional potential associated with microbial C metabolism (Douglas et al., 2020).

### 2.5 Statistical analysis and bioinformatic processing

Data were analyzed by IBM SPSS 22.0. The distribution of environmental variables was checked and data were transformed if necessary. One-way analysis of variance and Duncan’s multiple range test for multiple comparisons were conducted to assess significant differences in soil properties, bacterial diversity, and community characteristics.

The following analysis was conducted using R 4.3.3. Soil bacterial diversity was characterized by taxonomic (richness index) and phylogenetic diversities by employing the diversity function within the vegan package. Using taxonomic and phylogenetic diversities, an integrated index reflecting soil bacterial diversity was established based on principal component analysis. This index was characterized by the score of the first axis, which explained 97.4% variance as it was positively related with taxonomic and phylogenetic diversities (*p* < 0.05; Supplementary Figure S2). Soil bacterial community structure was characterized by the Jaccard dissimilarity coefficient by employing the vegdist function within the vegan package. The relative contribution of nestedness (richness difference) to soil bacterial community structure was evaluated using beta.div.comp function in the adespatial package (Legendre, 2014). A soil bacterial co-occurrence network was constructed and filtered using a relevance threshold of 0.90 and a significance level set at 0.01, which were determined by random matrix theory using untransformed abundances of OTUs (Deng et al., 2012). The subgraph function of the igraph package was used to preserve OTUs in each sample, separate the sub-network and calculate the topological features of each sub-network. Topological features of soil bacterial co-occurrence network included node number (number of nodes), edge number (number of connections), positive/negative edge number (ratio of positive to negative connections), average degree (mean connection of each node), and degree centralization (extended from the corresponding node-level significance). Using topological features, an integrated index reflecting network complexity was established based on principal component analysis. This index was characterized by the score of the first axis, which explained 81.2% variance as it was positively related with topological features (*p* < 0.05; Supplementary Figure S3). The stability of soil bacterial co-occurrence network was characterized by robustness and vulnerability indices (Yuan et al., 2021). According to the criterion suggested by Ning et al. (2019), relative contributions of deterministic and stochastic assembly processes of soil bacterial communities were quantified by phylogenetic normalized stochasticity ratio (pNST). Low pNST value indicated a strong influence of deterministic processes, while high pNST value indicated a strong influence of stochastic processes.

Next, factors affecting soil bacterial community structure were recognized. The relative importance of soil properties to bacterial diversity and network complexity was assessed through random forest analysis using the rfPermute package. Pearson correlation analysis was performed to evaluate the links between soil properties, bacterial community characteristics, microbial C metabolism, and SOC decomposition. Linear relationship analysis was implemented to assess the links between microbial C metabolism and SOC decomposition. Module construction was performed using Gephi, in which three modules were identified in which node number was more than 10. Using topological features of modules, an integrated index reflecting species association within three modules was established based on principal component analysis. Microbial C metabolism of modules and taxa within modules was assessed by functional prediction results related to the encoding of enzymes (*β*-1,4-glucosidase, EC: 3.2.1.21; cellobiohydrolase, EC:3.2.1.91; peroxidase, EC: 1.11.1.15; phenol oxidase, EC: 1.14.18.1) according to the standard of Waldrop et al. (2000) and Hasanuzzaman et al. (2020). The effect of soil properties, the richness index of keystone taxa and species association within keystone module, microbial C metabolism, and SOC decomposition was constructed through partial least squares path modeling analysis (PLS-PM).

## 3 Result

### 3.1 Variations in microbial C metabolism and SOC decomposition

**Figure 1:**
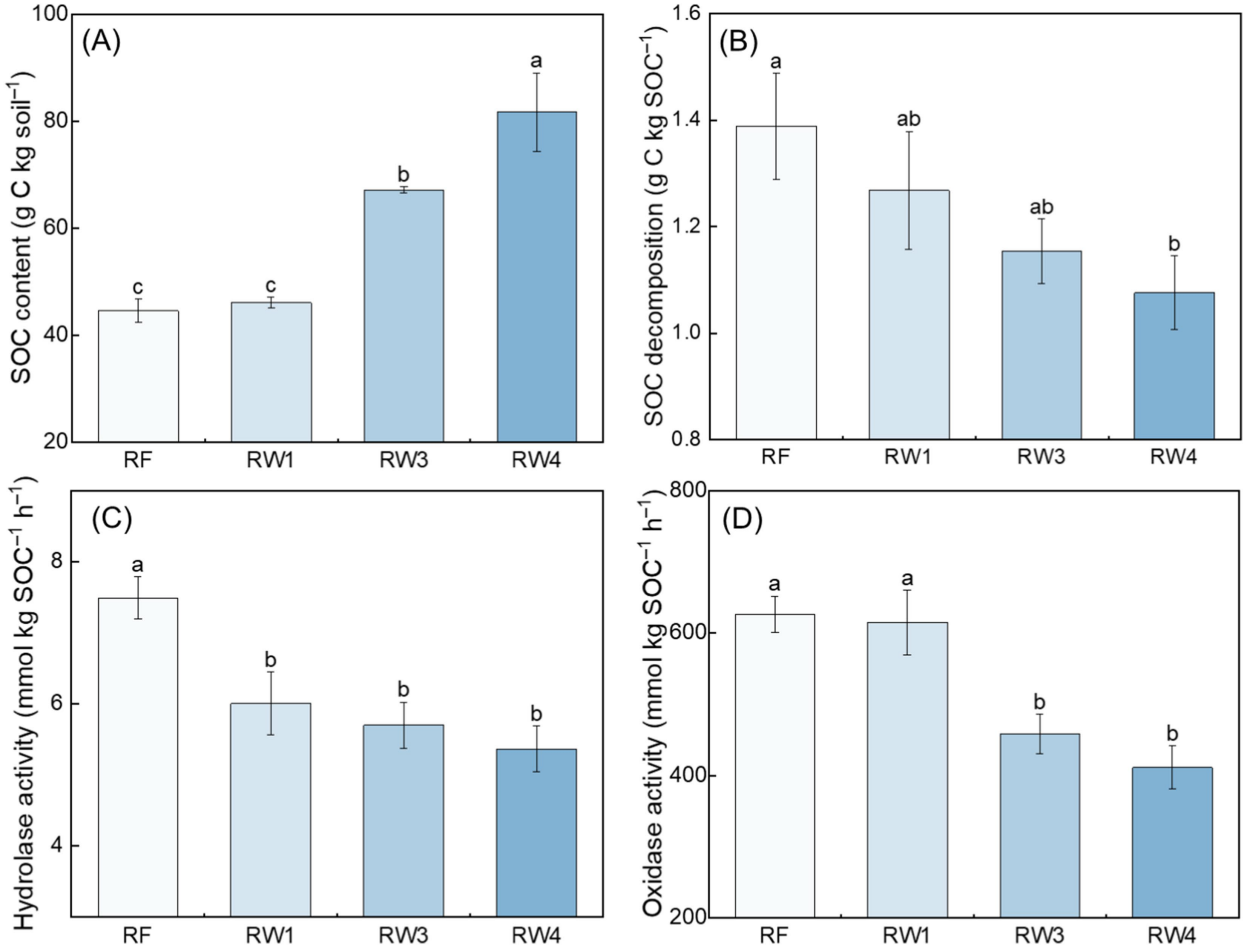
Effects of wetland restoration after agricultural abandonment on the **(A)** content and **(B)** decomposition rate of SOC and **(C,D)** microbial C metabolism. In panels **(C,D)**, the activities of hydrolase and oxidase represent microbial C metabolism. Vertical bars denote the standard errors of the mean (*n* = 5). Different letters indicate significant differences between treatments at *p* < 0.05.

Compared with RF treatment, SOC content increased under RW3 and RW4 treatments by 50.9–83.6%, while SOC decomposition rate decreased under RW4 treatment by 22.5% (*p* < 0.05; Figures 1A,B). Wetland restoration decreased microbial C metabolism characterized by the decreased hydrolase activity under all restored treatments by 19.8–28.4% and the decreased oxidase activity under RW3 and RW4 treatments by 26.9–34.4% (*p* < 0.05; Figures 1C,D).

### 3.2 Soil bacterial community succession

**Figure 2:**
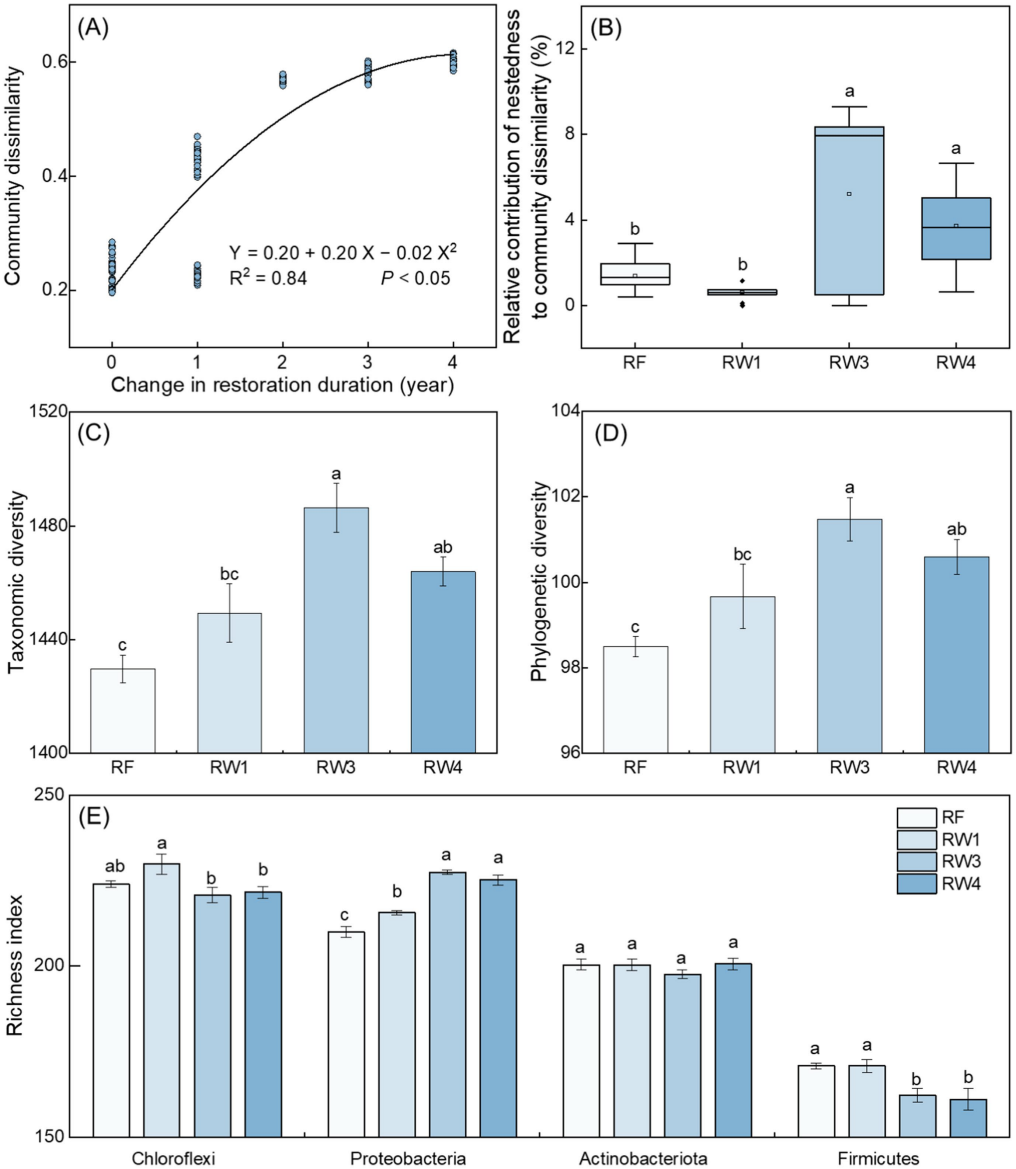
Effects of wetland restoration after agricultural abandonment on the succession of soil bacterial communities. In panel **(A)**, regression analysis shows the relationship between community dissimilarity and change in restoration duration. In panel **(B)**, nestedness to community dissimilarity represent richness difference on the variation of community structure. The boxplots include each box showing mean, median, quartiles, and values within 1.5-times the interquartile range. Different letters indicate significant differences between treatments at *p* < 0.05. In panels **(C–E)**, taxonomic diversity is characterized by richness index; the richness index of main phylum is greater than 150. Vertical bars denote the standard errors of the mean (*n* = 5). Different letters indicate significant differences between treatments at *p* < 0.05.

Soil bacterial community dissimilarity increased with the increase of restoration duration (*p* < 0.05; Figure 2A), indicating that wetland restoration observably drove soil bacterial community succession. Wetland restoration increased the relative contribution of nestedness (richness difference) in the variation of soil bacterial communities under RW3 and RW4 treatments (*p* < 0.05; Figure 2B).

Compared with RF treatment, soil bacterial taxonomic (characterized by richness index) and phylogenetic diversities increased under RW3 and RW4 treatments by 2.39–3.96% and 2.13–3.02%, respectively (*p* < 0.05; Figures 2C,D). Pearson correlation displayed that soil Fe^2+^/Fe^3+^ and the contents of SOC, and DOC were positively related with bacterial diversity (*p* < 0.05; Supplementary Figure S4). Random forest analysis suggested that DOC and SOC contents were the most important factors on bacterial diversity (*p* < 0.05; Supplementary Figure S5A).

Specifically, wetland restoration increased the richness index of Proteobacteria under all restored treatments by 2.67–8.29%, while decreased the richness index of Firmicutes under RW3 and RW4 treatments by 5.04–5.74% (*p* < 0.05; Figure 2E). Random forest analysis suggested that DOC was the most important factor on the richness index of Proteobacteria, while soil Fe^2+^/Fe^3+^ was the most important factor on the richness index of Firmicutes (*p* < 0.05; Supplementary Figure S6).

### 3.3 Variations in soil bacterial co-occurrence network

**Figure 3:**
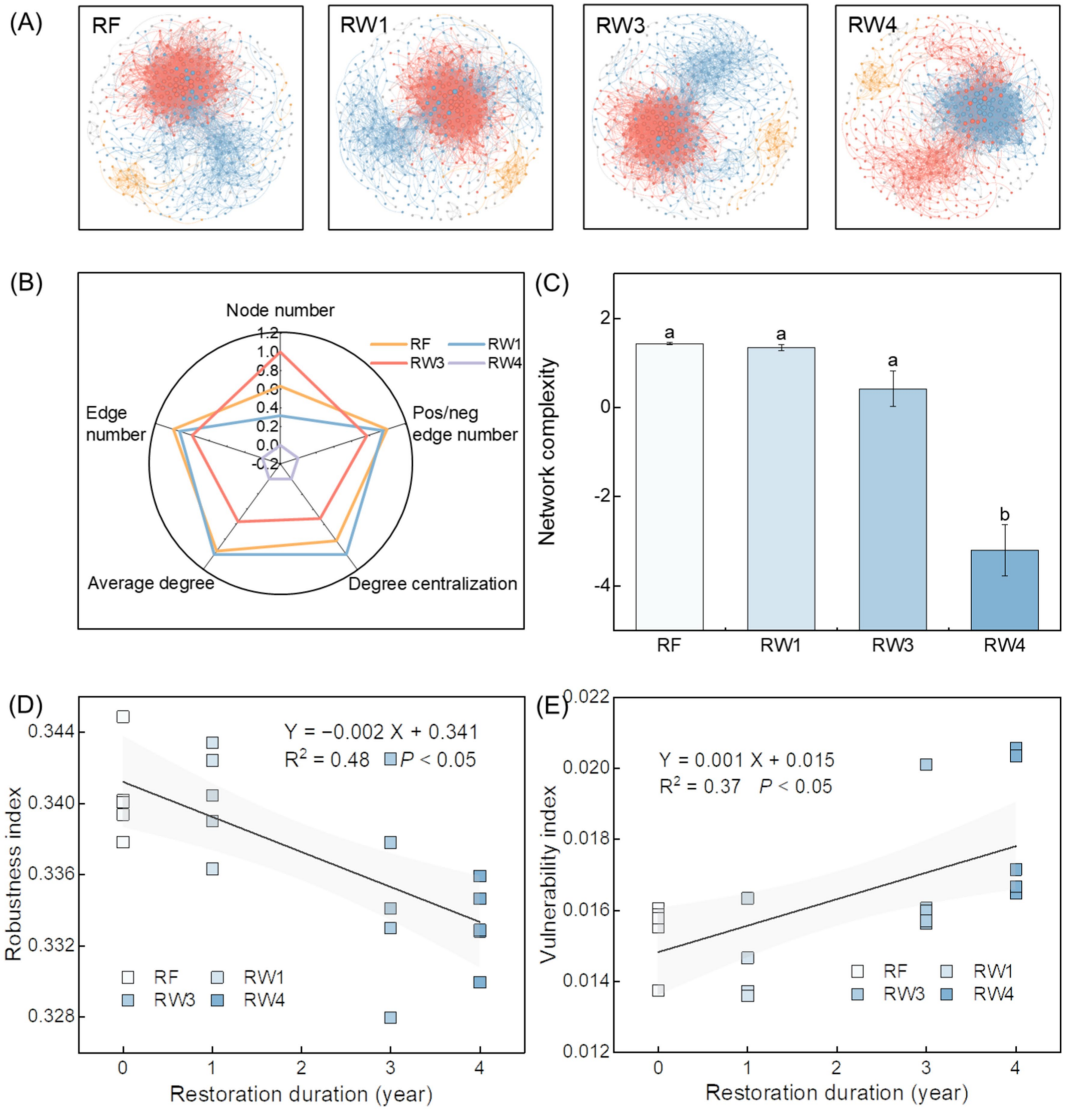
Effects of wetland restoration after agricultural abandonment on complexity and stability of soil bacterial co-occurrence network. In panel **(A)**, the nodes and edges are colored according to the modules they belong to, and their sizes are proportional to the degree of the corresponding operational taxonomic units (OTUs). In panel **(B)**, species association is characterized by the topological features including node number, edge number, pos/neg edge number (positive/negative edge number), average degree, and degree centralization. In panel **(C)**, network complexity is characterized by the first axis of topological features based on principal component analysis. Vertical bars denote the standard errors of the mean (*n* = 5). Different letters indicate significant differences between treatments at *p* < 0.05. In panels **(D,E)**, robustness and vulnerability indices represent network stability. Shaded areas represent the 95% confidence interval of the regression line.

Wetland restoration changed soil bacterial co-occurrence network (Figure 3A). Specifically, wetland restoration decreased node number, edge number, and positive/negative edge number under RW4 treatment by 2.52%, 11.9%, and 6.37%, respectively, average degree and degree centralization under RW3 and RW4 treatments by 3.92–9.62% and 2.28–6.34%, respectively (*p* < 0.05; Figure 3B; Supplementary Table S2). Given the above, wetland restoration decreased network complexity of soil bacterial communities under RW4 treatment (*p* < 0.05; Figure 3C). Pearson correlation showed that soil Fe^2+^/Fe^3+^, the contents of SOC, DOC and inorganic N, SOC/TN, and SOC/TP were negatively correlated with network complexity (*p* < 0.05; Supplementary Figure S4). Random forest analysis suggested that soil Fe^2+^/Fe^3+^ was the most important factor on network complexity (*p* < 0.05; Supplementary Figure S5B).

Compared with RF treatment, network stability of soil bacterial communities decreased by the decreased robustness index while the increased vulnerability index with the increase of restoration duration (*p* < 0.05; Figures 3D,E). The robustness index was positively related with network complexity, while the vulnerability index was negatively related with network complexity (*p* < 0.05; Supplementary Figure S7), indicating that there was a positive relationship between network complexity and stability of soil bacterial communities.

**Figure 4:**
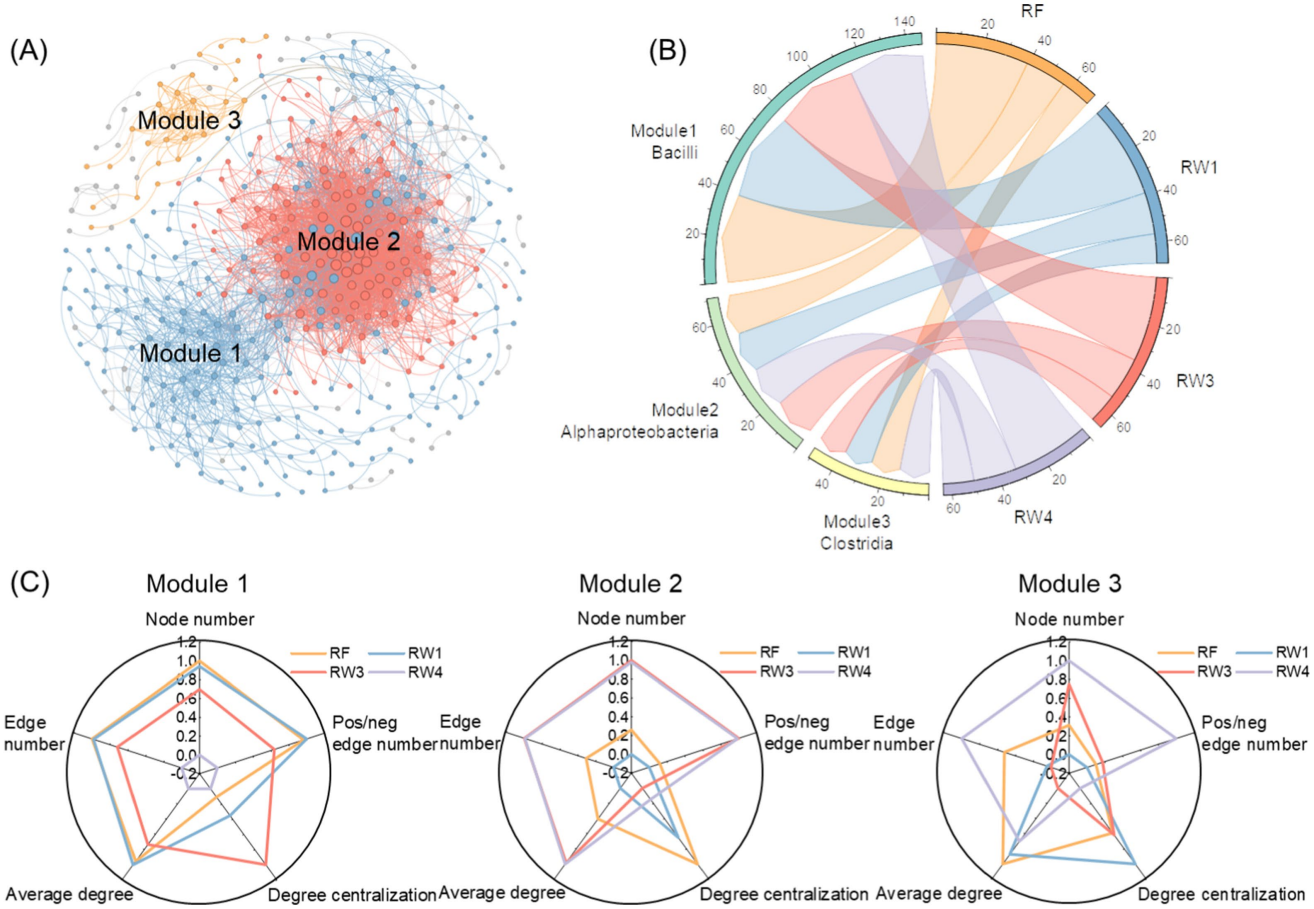
Effects of wetland restoration after agricultural abandonment on species associations within modules in soil bacterial co-occurrence network. In panel **(A)**, the nodes and edges are colored according to the modules they belong to, and their sizes are proportional to the degree of the corresponding OTUs. Modules were identified in which node number was more than 10. In panel **(B)**, the taxa at the class level with highest average richness index were identified. In panel **(C)**, radar plot shows species associations within modules characterized by the topological features including node number, edge number, pos/neg edge number (positive/negative edge number), average degree, and degree centralization.

There were three modules among soil bacterial co-occurrence network (Figure 4A). Bacilli had the most degree and the largest number of high within-module connectivities in module 1, its richness index decreased under RW4 treatment by 16.4% (*p* < 0.05; Figure 4B; Supplementary Tables S3–S5). Acidobacteriae had the most degree and the largest number of high within-module connectivities in module 2, its richness index increased under RW3 and RW4 treatments by 7.14% (*p* < 0.05). Clostridia had the most degree and the largest number of high within-module connectivities in module 3, its richness index did not alter significantly under all restored treatments. Among three modules, node number in module 1 decreased under RW4 treatment by 10.8%, while edge number, positive/negative edge number, and average degree in module 1 under RW3 and RW4 treatments reduced by 4.86–17.2%, 4.52–13.3%, and 1.67–7.23%, respectively (*p* < 0.05; Figure 4C; Supplementary Table S2). In contrast, node number, edge number, and positive/negative edge number in module 2 increased under RW3 and RW4 treatments by 5.72–5.88%, 8.74–8.82%, and 6.03–6.11%, respectively (*p* < 0.05); node number in module 3 also increased under RW4 treatment by 3.63% (*p* < 0.05).

### 3.4 Soil bacterial community assembly

Wetland restoration increased deterministic processes of soil bacterial community assembly under RW1 treatment characterized by decreased pNST value (*p* < 0.05; Supplementary Figure S8A). There were significant relationships between pNST value and variations in soil anaerobic condition (characterized by soil Fe^2+^/Fe^3+^), labile C supply (characterized by DOC content) and species association (characterized by topological features of co-occurrence network) (*p* < 0.05; Supplementary Figures S8B–D).

### 3.5 Linking soil bacterial co-occurrence network with C metabolism and SOC decomposition

**Figure 5:**
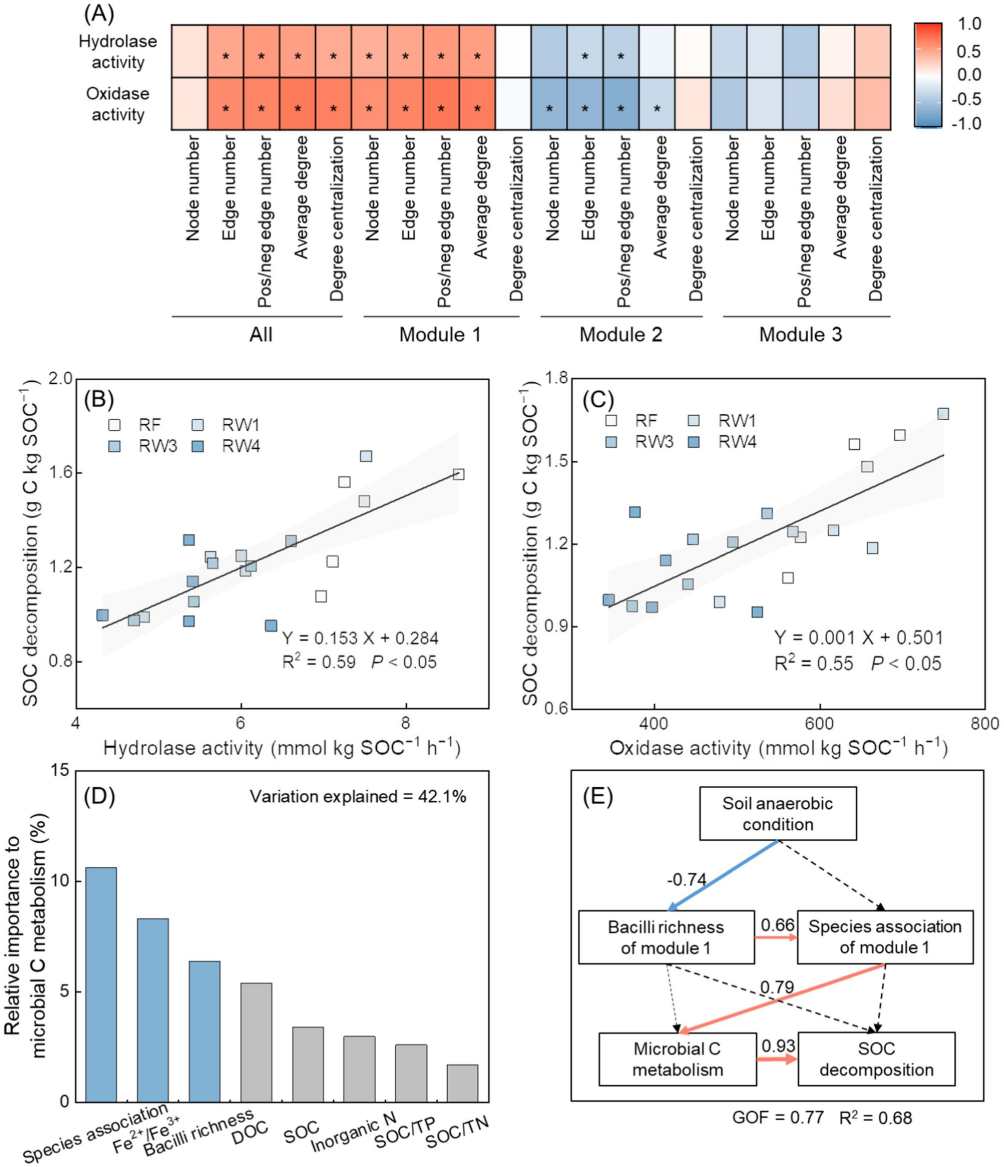
Effects of soil properties and species associations on microbial C metabolism and SOC decomposition during wetland restoration after agricultural abandonment. In panel **(A)**, the heatmap shows the relationships between species associations within overall network and each module and microbial C metabolism. Species associations are characterized by the topological features including node number, edge number, pos/neg edge number (positive/negative edge number), average degree, and degree centralization. The activities of hydrolase and oxidase represent microbial C metabolism. Significance level is *p < 0.05. In panels **(B,C)**, linear regression analysis shows the relationships between microbial C metabolism and SOC decomposition. Microbial C metabolism is characterized by the activities of hydrolase and oxidase. Shaded areas represent the 95% confidence interval of the regression line. In panel **(D)**, random forest analysis shows the relative importance of soil properties, species association within keystone module, and richness of keystone taxa in keystone module on microbial C metabolism. Species association within module 1 is characterized by the first axis of topological features based on principal component analysis; Bacilli richness in module 1 by richness index; microbial C metabolism is characterized by the first axis of the activities of hydrolase and oxidase based on principal component analysis. Blue columns denote significant influences of parameters at *p* < 0.05; grey columns denote insignificant influences of parameters at *p* > 0.05. Fe^2+^/Fe^3+^, the ratio of soil ferrous iron to ferric iron; SOC, soil organic carbon; DOC, dissolved organic carbon; Inorganic N, inorganic nitrogen; SOC/TN, the ratio of soil organic carbon to total nitrogen; SOC/TP, the ratio of soil organic carbon to total phosphorus. In panel **(E)**, partial least squares path modeling (PLS-PM) analysis shows the effects of soil anaerobic condition, species association within keystone module, richness of keystone taxa in keystone module, and microbial C metabolism on SOC decomposition. Values adjoining the arrows indicate standardized path coefficients and arrow width is proportional to the strength of the relationship. Red and blue arrows represent positive and negative relationships, respectively. Solid and dotted arrows represent significant (*p* < 0.05) and insignificant (*p* > 0.05) relationships, respectively. The model is evaluated using the goodness of fit (GOF) statistic. R^2^ indicates the variance of soil anaerobic condition, Bacilli richness of module 1, and species association within module 1 accounted for by the model. Soil anaerobic condition is characterized by soil Fe^2+^/Fe^3+^; Bacilli richness of module 1 is characterized by richness index; species association within module 1 is characterized by the first axis of topological features based on principal component analysis; microbial C metabolism is characterized by the activities of hydrolase and oxidase.

There were positive relationships between the activities of hydrolase and oxidase and the decomposition rate of SOC (*p* < 0.05; Figures 5B,C). The activities of hydrolase and oxidase were positively related with the topological features of bacterial co-occurrence network, with stronger relationships to module 1 while extremely weak to module 2 and module 3 (*p* < 0.05; Figure 5A). The activities of hydrolase and oxidase were also positively related with the richness index of Bacilli in module 1 (*p* < 0.05; Supplementary Figure S9). Functional prediction results showed that in module 1, the abundance of hydrolase decreased under RW3 and RW4 treatments by 68.6–72.6%, and the abundance of oxidase lowered under all restored treatments by 2.69–67.2% (*p* < 0.05; Supplementary Figure S10). Among the taxa at the class level in module 1, Bacilli had the most encoded enzymes for C metabolism (Supplementary Table S6). Random forest analysis revealed that soil Fe^2+^/Fe^3+^, the richness index of Bacilli and species association in module 1 (characterized by the first axis of topological features explained 77.3% variance) were the important factors influencing microbial C metabolism (*p* < 0.05; Figure 5D). The PLS-PM results showed that lower species association within module 1 induced by lower richness index of Bacilli indirectly influenced on microbial C metabolism under stronger anaerobic condition, which weakened SOC decomposition during wetland restoration (*p* < 0.05; Supplementary Figure S11; Figure 5E).

## 4 Discussion

### 4.1 Wetland restoration increased soil bacterial taxonomic and phylogenetic diversities

Wetland restoration observably drove soil bacterial community succession (Figure 2A), which was consistent with previous studies (Ge et al., 2023; Meng et al., 2024). The relative contribution of nestedness in soil bacterial community succession dramatically increased (Figure 2B), indicating that richness difference driven by environmental filtering played a more vital role during wetland restoration. Wetland restoration significantly increased taxonomic (richness) and phylogenetic diversities (Figures 2C,D), suggesting that niche space was expanded to allow for the existence of many species (Larkin and Martiny, 2017). This might be because more supply of available nutrients (increased DOC content) induced higher soil bacterial diversity during wetland restoration (Supplementary Figure S5A), which aligned with the results of Li et al. (2021) in the Yellow River Delta. Specifically, increased DOC content promoted the increase of Proteobacteria richness index during wetland restoration (Supplementary Figure S6A; Figure 2E), which might be because Proteobacteria could consume labile organic substrates and are classified as copiotrophic bacteria (Fierer et al., 2007). In contrast, stronger soil anaerobic condition (characterized by increased Fe^2+^/Fe^3+^) induced the decrease of Firmicutes richness index during wetland restoration (Supplementary Figure S6B; Figure 2E), which might be because Firmicutes are assigned to the aerobic bacteria (Reddy et al., 2011). Liu et al. (2021) also suggested that wetland soils exhibited the lower proliferation of aerobic bacteria under flood condition compared with drought condition. Thus, our results suggested that although more labile C supply induced higher soil bacterial diversity, stronger anaerobic condition inhibited the growth of aerobic species especially Firmicutes during wetland restoration.

### 4.2 Wetland restoration decreased network complexity and stability of soil bacterial communities

Wetland restoration notably decreased network complexity (Figure 3C), which was positively correlated with network stability of soil bacterial communities (Supplementary Figure S7; Figures 3D,E). The result was aligned with the results in natural ecosystems (Gao et al., 2022; Zhang C. et al., 2024), while was inconsistent with some results of theoretical models and culture experiments (Coyte et al., 2015; Yonatan et al., 2022). This might be because not all individual microorganisms had the ability to synthesize the substances they need, they could cooperate to resist environmental pressures and disturbances by close interactions and synchronous responses in natural ecosystems (Valdivia et al., 2012; Wang J. et al., 2023). In this study, stronger soil anaerobic condition drove the decrease of species association especially positive association in module 1 during wetland restoration (Supplementary Figure S5B; Figures 3B, 4C). This is primarily because wetland restoration decreased the richness index of Bacilli in module 1 (Figure 4B; Supplementary Table S3), which were most important keystone taxa at the class level associated with the highest richness index, the most degree, and the largest number of high within-module connectivities (Supplementary Tables S3–S5). Shafi et al. (2017) suggested that Bacilli affiliate to Firmicutes phylum, tend to occupy aerobic niches, and can produce a lot of secondary metabolites (such as volatile organic compounds) that coordinate mutualistic and syntrophic interactions through quorum sensing mechanism (Sarsan et al., 2021). Wetland restoration increased deterministic processes of microbial community assembly that was significantly correlated with the variation of soil anaerobic condition (Supplementary Figures S8A,B), also confirming the important role of environmental filtering in soil bacterial co-occurrence network.

### 4.3 Wetland restoration reduced C metabolism and SOC decomposition due to decreased species association within Bacilli-dominated keystone module

Wetland restoration markedly decreased microbial C metabolism and SOC decomposition (Figures 1B–D), which aligned with previous studies (Tang et al., 2024; Zhang S. et al., 2024). Microbial C metabolism is a cascading SOC decomposition process driven by diverse bacteria with distinct functions accomplished through metabolic chains (Schimel and Schaeffer, 2012). In this process, modules formed by specific bacteria play pivotal roles in initiating metabolic cascades or mediating the transfer of metabolites, thereby exerting more significant influences on SOC decomposition than the entire community (Banerjee et al., 2018; Bian et al., 2022). We found that only module 1 of soil bacterial communities exhibited significantly lower microbial C metabolism during wetland restoration (Supplementary Figure S10), indicating that module 1 played a key role in reducing microbial C metabolism.

We observed that only species association within module 1 was positively correlated with the activities of hydrolase and oxidase and SOC decomposition (Figure 5A). Among the taxa in module 1, Bacilli class affiliating to Firmicutes phylum, were most important keystone taxa and had the most encoded enzymes for C metabolism (Supplementary Tables S3–S6). Panosyan et al. (2018) also suggested that Bacilli exhibit different metabolic capabilities for degrading diverse organic molecules by secreting catalase and oxidase, in which low molecular organic acids are produced as metabolic byproducts to support downstream microorganisms, thus promote microbial C metabolism (Ruiz-Villafán et al., 2022). Therefore, the decrease richness index of Bacilli in module 1 might inhibit catabolism and metabolite exchange through species association (Figure 5E). Overall, our results suggested that wetland restoration decreased positive association within Bacilli-dominated keystone module under stronger anaerobic condition, thereby mitigating microbial C metabolism and SOC decomposition during wetland restoration.

### 4.4 Implications for future research

Our results evidenced that wetland restoration could achieve the dual benefits of diversity conservation and SOC sequestration, and emphasized the important role of species association of keystone module in microbial C metabolism. Given that microbial activities reflect the mutual influences of complex environments, further experimental validation is necessary to assess their individual roles. In addition, the biochemical reactions facilitated by enzymes may not represent the full range of metabolic activities occurring in soil bacterial communities, therefore, employing more sophisticated techniques, like metagenomics, is recommended to investigate the critical functional groups that affect SOC decomposition. This would help enhance our understanding of C fate in soils, and improve the ability to integrate microbial ecology into global C cycle model.

## 5 Conclusion

Our study reported that wetland restoration increased soil bacterial diversity and demonstrated that lower species association within keystone module reduced microbial C metabolism and SOC decomposition amid stronger anaerobic condition. We found that wetland restoration increased soil bacterial taxonomic and phylogenetic diversities, in which increased nestedness (richness) drove the variation of soil bacterial community structure. However, soil Fe^2+^/Fe^3+^ exhibited the most important impact on decreased Firmicutes richness and network complexity. Specifically, wetland restoration decreased positive association, especially in keystone module mainly composed of aerobic Bacilli, which was unfavorable for microbial C metabolism. Therefore, maintaining moderate standing water depth (20–40 cm) could preserve anaerobic condition that inhibit the proliferation and association of aerobic species in keystone module, thereby reducing SOC decomposition and enhancing SOC sequestration during wetland restoration. In the future, similar experiments across different climate zones and wetland types are required to integrate microbial co-occurrence network into C sequestration monitoring frameworks.

## Data Availability

The datasets presented in this study can be found in online repositories. The names of the repository/repositories and accession number(s) can be found at: https://www.ncbi.nlm.nih.gov/, PRJNA1213004.
