# Supplementary material for: Wetland restoration suppresses microbial carbon metabolism by altering keystone species interactions
Source: Front Microbiol. 2025 Apr 30;16:1570703. doi: 10.3389/fmicb.2025.1570703 (PMC12075313; doi:10.3389/fmicb.2025.1570703)
Supplement: Supplementary file 1 [file Data_Sheet_1.docx]

**Appendix A. Supplementary data**

**Table S1.** Effects of wetland restoration after agricultural abandonment on soil properties.

|  | RF | RW1 | RW3 | RW4 |
| --- | --- | --- | --- | --- |
| pH | 6.04 ± 0.05a | 6.02 ± 0.06a | 5.98 ± 0.02a | 5.96 ± 0.03a |
| Fe^2+^/Fe^3+^ | 0.20 ± 0.01c | 0.22 ± 0.01c | 0.55 ± 0.03b | 0.64 ± 0.03a |
| SOC/TN | 13.0 ± 0.75a | 13.4 ± 0.80a | 14.0 ± 0.19a | 15.0 ± 1.23a |
| SOC/TP | 76.7 ± 6.57b | 76.0 ± 6.17b | 86.1 ± 4.71ab | 105 ± 9.40a |
| DOC | 55.4 ± 1.64c | 62.7 ± 3.54c | 95.3 ± 4.41b | 125 ± 5.03a |
| Inorganic N | 8.48 ± 0.44b | 9.19 ± 0.69b | 12.0 ± 0.90a | 12.1 ± 0.34a |

RF, paddy fields; RW1, wetland restored from paddy fields for 1 year; RW3, wetland restored from paddy fields for 3 years; RW4, wetland restored from paddy fields for 4 years.

Fe^2+^/Fe^3+^, the ratio of soil ferrous iron to ferric iron; SOC/TN, the ratio of soil organic carbon to total nitrogen; SOC/TP, the ratio of soil organic carbon to total phosphorus; DOC, dissolved organic carbon; Inorganic N, inorganic nitrogen.

Means ± standard error (*n* = 5). Different letters within the same column indicate significant differences between treatments at *P* < 0.05.

**Table S2.** Effects of wetland restoration after agricultural abandonment on the topological features of soil bacterial co-occurrence network.

|  | Topological feature | RF | RW1 | RW3 | RW4 |
| --- | --- | --- | --- | --- | --- |
| All | Node number | 399 ± 1.46ab | 394 ± 2.20bc | 405 ± 2.35a | 389 ± 2.65c |
|  | Edge number | 3984 ± 10.2a | 3952 ± 6.25a | 3884 ± 48.3a | 3512 ± 52.1b |
|  | Pos/neg edge number | 2.27 ± 0.01a | 2.27 ± 0.01a | 2.24 ± 0.01a | 2.13 ± 0.03b |
|  | Average degree | 20.0 ± 0.08a | 20.1 ± 0.10a | 19.2 ± 0.14b | 18.0 ± 0.15c |
|  | Degree centralization | 0.24 ± 0.00b | 0.24 ± 0.00a | 0.23 ± 0.00c | 0.22 ± 0.00d |
| Module 1 | Node number | 159 ± 0.86a | 158 ± 0.49a | 154 ± 1.92a | 142 ± 2.61b |
|  | Edge number | 2899 ± 10.1a | 2890 ± 6.58a | 2758 ± 45.9b | 2399 ± 50.9c |
|  | Pos/neg edge number | 1.97 ± 0.01a | 1.98 ± 0.01a | 1.88 ± 0.02b | 1.71 ± 0.04c |
|  | Average degree | 36.4 ± 0.09a | 36.5 ± 0.11a | 35.8 ± 0.26b | 33.8 ± 0.13c |
|  | Degree centralization | 0.44 ± 0.00b | 0.45 ± 0.00ab | 0.45 ± 0.00a | 0.44 ± 0.00b |
| Module 2 | Node number | 126 ± 0.86b | 123 ± 0.58c | 133 ± 0.20a | 133 ± 0.32a |
|  | Edge number | 506 ± 5.45b | 486 ± 9.56c | 550 ± 0.20a | 550 ± 0.49a |
|  | Pos/neg edge number | 8.00 ± 0.05b | 7.93 ± 0.11b | 8.49 ± 0.00a | 8.48 ± 0.01a |
|  | Average degree | 8.04 ± 0.13ab | 7.89 ± 0.16b | 8.26 ± 0.01a | 8.27 ± 0.01a |
|  | Degree centralization | 0.22 ± 0.00a | 0.22 ± 0.00a | 0.22 ± 0.00a | 0.22 ± 0.00a |
| Module 3 | Node number | 60.6 ± 0.87bc | 59.6 ± 0.60c | 62.0 ± 0.32ab | 62.8 ± 0.20a |
|  | Edge number | 181 ± 2.06ab | 177 ± 1.75b | 176 ± 3.54b | 185 ± 1.40a |
|  | Pos/neg edge number | 11.0 ± 0.14a | 10.8 ± 0.12a | 11.2 ± 0.08a | 13.4 ± 2.00a |
|  | Average degree | 5.96 ± 0.06a | 5.93 ± 0.04a | 5.68 ± 0.10b | 5.88 ± 0.03a |
|  | Degree centralization | 0.19 ± 0.00ab | 0.19 ± 0.00a | 0.19 ± 0.00ab | 0.18 ± 0.00b |

Pos/neg edge number, positive/negative edge number.

RF, paddy fields; RW1, wetland restored from paddy fields for 1 year; RW3, wetland restored from paddy fields for 3 years; RW4, wetland restored from paddy fields for 4 years.

Means ± standard error (*n* = 5). Different letters within the same column indicate significant differences between treatments at *P* < 0.05.

**Table S3.** Effects of wetland restoration after agricultural abandonment on the richness index of taxa at the class level (richness index was more than 10) in modules of soil bacterial co-occurrence network.

| Module | Class | RF | RW1 | RW3 | RW4 |
| --- | --- | --- | --- | --- | --- |
| Module 1 | Bacilli | 39.0 ± 0.00a | 39.0 ± 0.00a | 36.0 ± 1.05a | 32.6 ± 1.99b |
|  | Anaerolineae | 21.0 ± 0.00a | 21.0 ± 0.00a | 19.6 ± 0.24b | 18.6 ± 0.40c |
|  | Thermoleophilia | 16.0 ± 0.00a | 16.0 ± 0.00a | 15.0 ± 0.32a | 13.0 ± 0.63b |
|  | Alphaproteobacteria | 13.0 ± 0.00a | 13.0 ± 0.00a | 12.4 ± 0.24ab | 12.2 ± 0.37b |
| Module 2 | Alphaproteobacteria | 17.4 ± 0.24b | 17.6 ± 0.24ab | 18.0 ± 0.00a | 18.0 ± 0.00a |
|  | Acidobacteriae | 16.8 ± 0.37b | 16.6 ± 0.24b | 18.0 ± 0.00a | 18.0 ± 0.00a |
|  | Vicinamibacteria | 15.8 ± 0.37b | 15.0 ± 0.55b | 17.0 ± 0.00a | 17.0 ± 0.00a |
|  | Gammaproteobacteria | 11.0 ± 0.55b | 11.8 ± 0.20ab | 12.0 ± 0.00a | 12.0 ± 0.00a |
| Module 3 | Clostridia | 12.8 ± 0.20a | 12.4 ± 0.24a | 12.4 ± 0.24a | 13.0 ± 0.00a |

RF, paddy fields; RW1, wetland restored from paddy fields for 1 year; RW3, wetland restored from paddy fields for 3 years; RW4, wetland restored from paddy fields for 4 years.

Means ± standard error (*n* = 5). Different letters within the same column indicate significant differences between treatments at *P* < 0.05.

**Table S4.** The taxa at the class level with degree more than 100 in modules.

| Module | Class | Degree |
| --- | --- | --- |
| Module 1 | Bacilli | 1862 |
|  | Thermoleophilia | 849 |
|  | Alphaproteobacteria | 545 |
|  | Anaerolineae | 441 |
|  | MB-A2-108 | 417 |
|  | Ktedonobacteria | 386 |
|  | Gammaproteobacteria | 228 |
|  | Gitt-GS-136 | 173 |
|  | Acidobacteriae | 132 |
|  | KD4-96 | 124 |
|  | Aminicenantia | 120 |
|  | Symbiobacteriia | 114 |
| Module 2 | Acidobacteriae | 569 |
|  | Vicinamibacteria | 216 |
| Module 3 | Clostridia | 122 |

**Table S5.** The classification of operational taxonomic units (OTUs) with within-module connectivities greater than 2 in modules at the class level.

| Module | OTU | within-module connectivities | Class |
| --- | --- | --- | --- |
| Module 1 | OTU6726 | 2.40 | MB-A2-108 |
|  | OTU6661 | 2.27 | MB-A2-108 |
|  | OTU10494 | 2.23 | Bacilli |
|  | OTU6988 | 2.13 | Aminicenantia |
|  | OTU6568 | 2.13 | Bacilli |
|  | OTU6368 | 2.13 | Bacilli |
|  | OTU13215 | 2.10 | Bacilli |
|  | OTU7443 | 2.06 | Thermoleophilia |
| Module 2 | OTU6455 | 3.24 | Acidobacteriae |
|  | OTU3377 | 2.90 | Acidobacteriae |
|  | OTU6565 | 2.79 | Acidobacteriae |
|  | OTU9467 | 2.68 | Acidobacteriae |
|  | OTU9585 | 2.45 | Acidobacteriae |
|  | OTU7411 | 2.45 | Acidobacteriae |
|  | OTU10224 | 2.23 | Acidobacteriae |
|  | OTU6518 | 2.23 | Acidobacteriae |
|  | OTU7204 | 2.00 | Acidobacteriae |
|  | OTU10439 | 2.00 | Acidobacteriae |
| Module 3 | OTU10631 | 2.15 | Thermoleophilia |
|  | OTU10244 | 2.15 | Clostridia |
|  | OTU7459 | 2.15 | Clostridia |

**Table S6.** The taxa at the class level in modules that contain 20 encoded enzymes associated with carbon metabolism.

| Module | Class | Encoded enzymes |
| --- | --- | --- |
| Module 1 | Bacilli | 183 |
|  | Anaerolineae | 79 |
|  | Ktedonobacteria | 73 |
|  | Thermoleophilia | 38 |
|  | Gammaproteobacteria | 37 |
|  | Alphaproteobacteria | 34 |
| Module 2 | Alphaproteobacteria | 71 |
|  | Vicinamibacteria | 62 |
|  | Acidobacteriae | 60 |
|  | Gammaproteobacteria | 42 |
|  | Actinobacteria | 21 |
| Module 3 | Acidobacteriae | 44 |
|  | Bacteroidia | 39 |
|  | Gammaproteobacteria | 31 |
|  | Thermoleophilia | 28 |
|  | Clostridia | 27 |

**Figure S1.** Linear regression analysis shows the relationships between the activities of hydrolases and oxidases and the first two components (Component 1 and Component 2) of multiple axes based on principal component analysis.

**
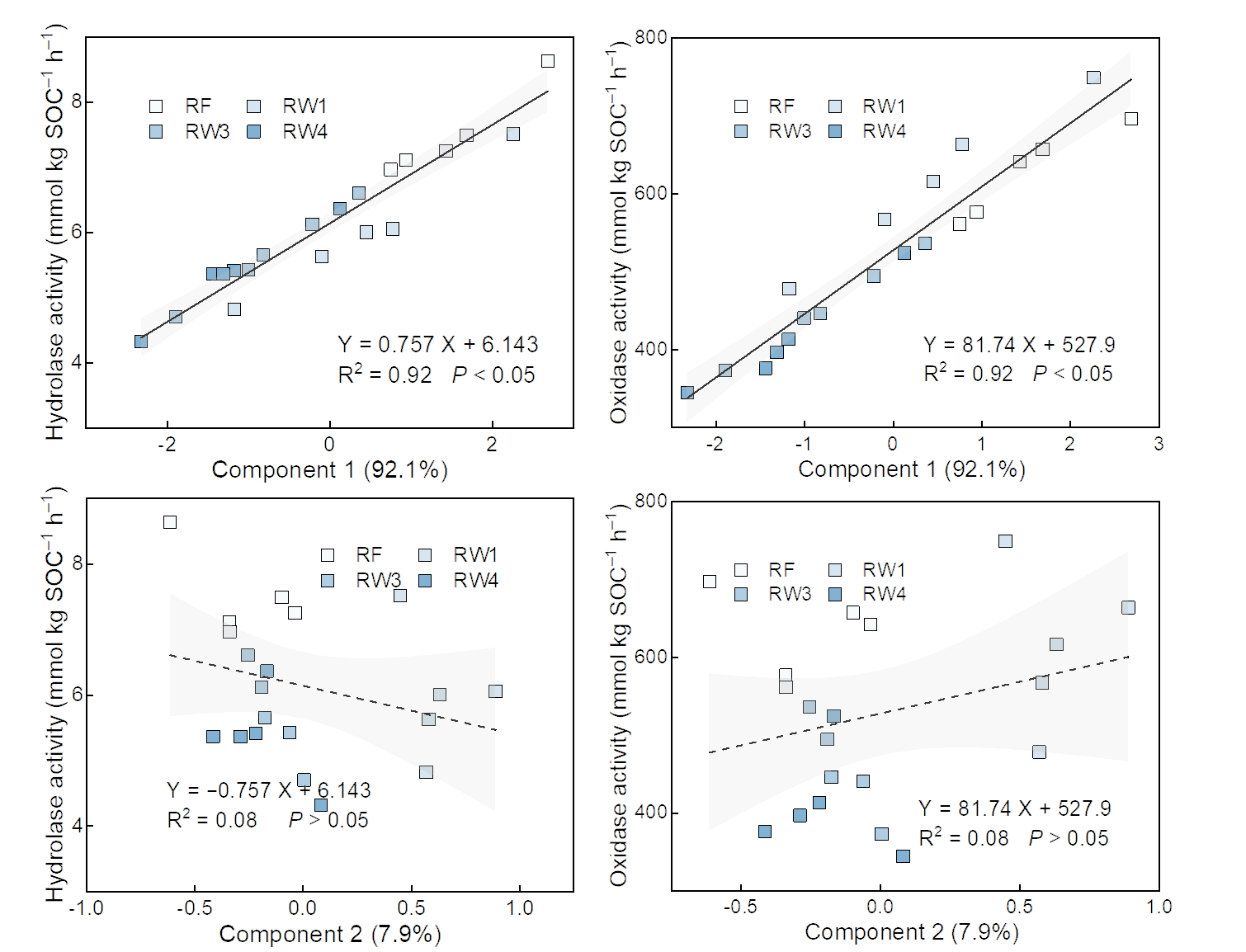
**

**Figure S2.** Linear regression analysis shows the relationships between soil bacterial (A) taxonomic and (B) phylogenetic diversities and the first component (Component 1) of multiple axes based on principal component analysis. Taxonomic diversity is characterized by the richness index.

**
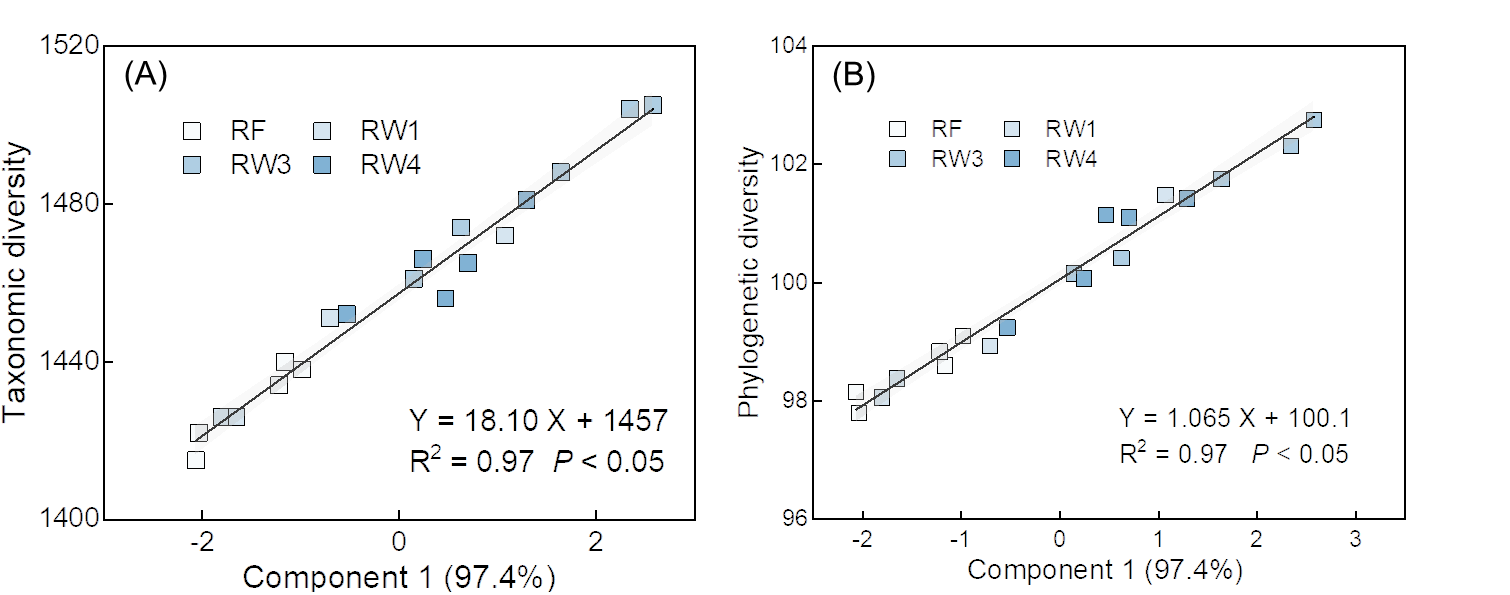
**

**Figure S3.** Pearson correlations between the topological features of soil bacterial co-occurrence network and different components (Com) of multiple axes based on principal component analysis. Significance level is **P* < 0.05.

**
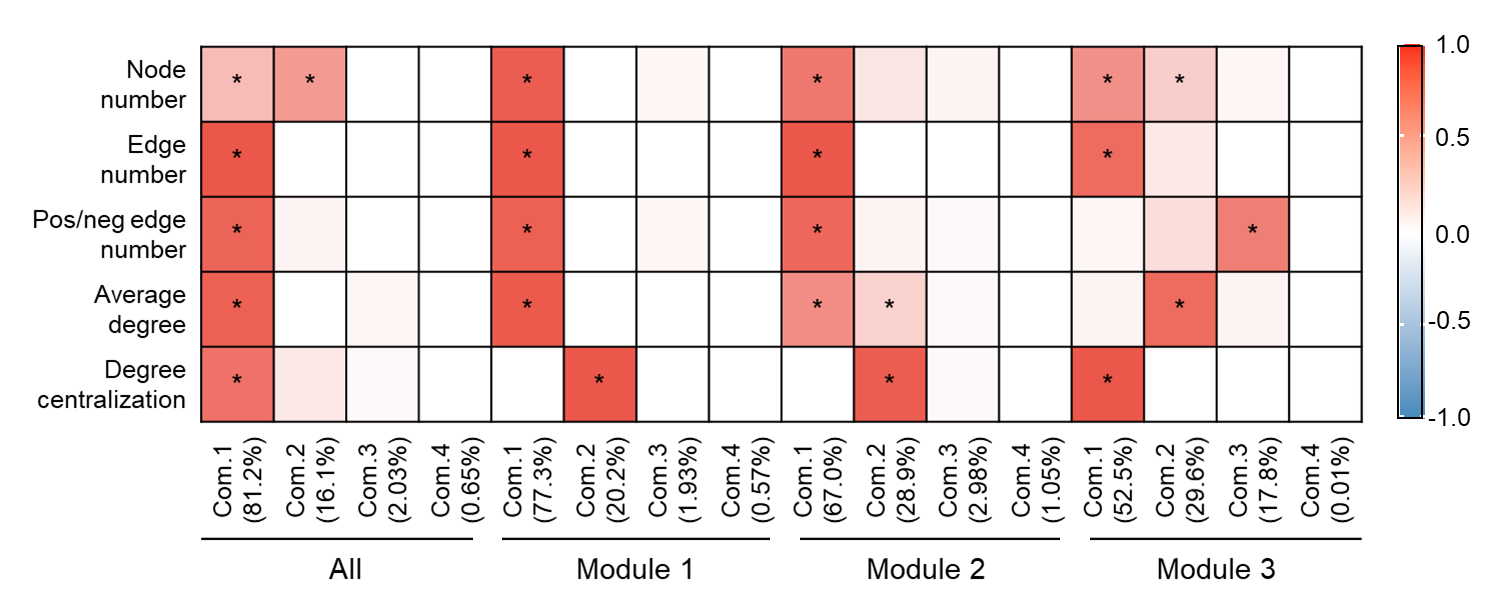
**

**Figure S4.** Pearson correlations between soil properties, bacterial diversity and network complexity. Bacterial diversity is characterized by the first axis of soil bacterial taxonomic and phylogenetic diversities based on principal component analysis. Taxonomic diversity is characterized by the richness index. Network complexity is characterized by the first axis of topological features of soil bacterial co-occurrence network based on principal component analysis. Fe^2+^/Fe^3+^, the ratio of soil ferrous iron to ferric iron; SOC, soil organic carbon; DOC, dissolved organic carbon; Inorganic N, inorganic nitrogen; SOC/TN, the ratio of soil organic carbon to total nitrogen; SOC/TP, the ratio of soil organic carbon to total phosphorus. Significance level is **P* < 0.05.

**
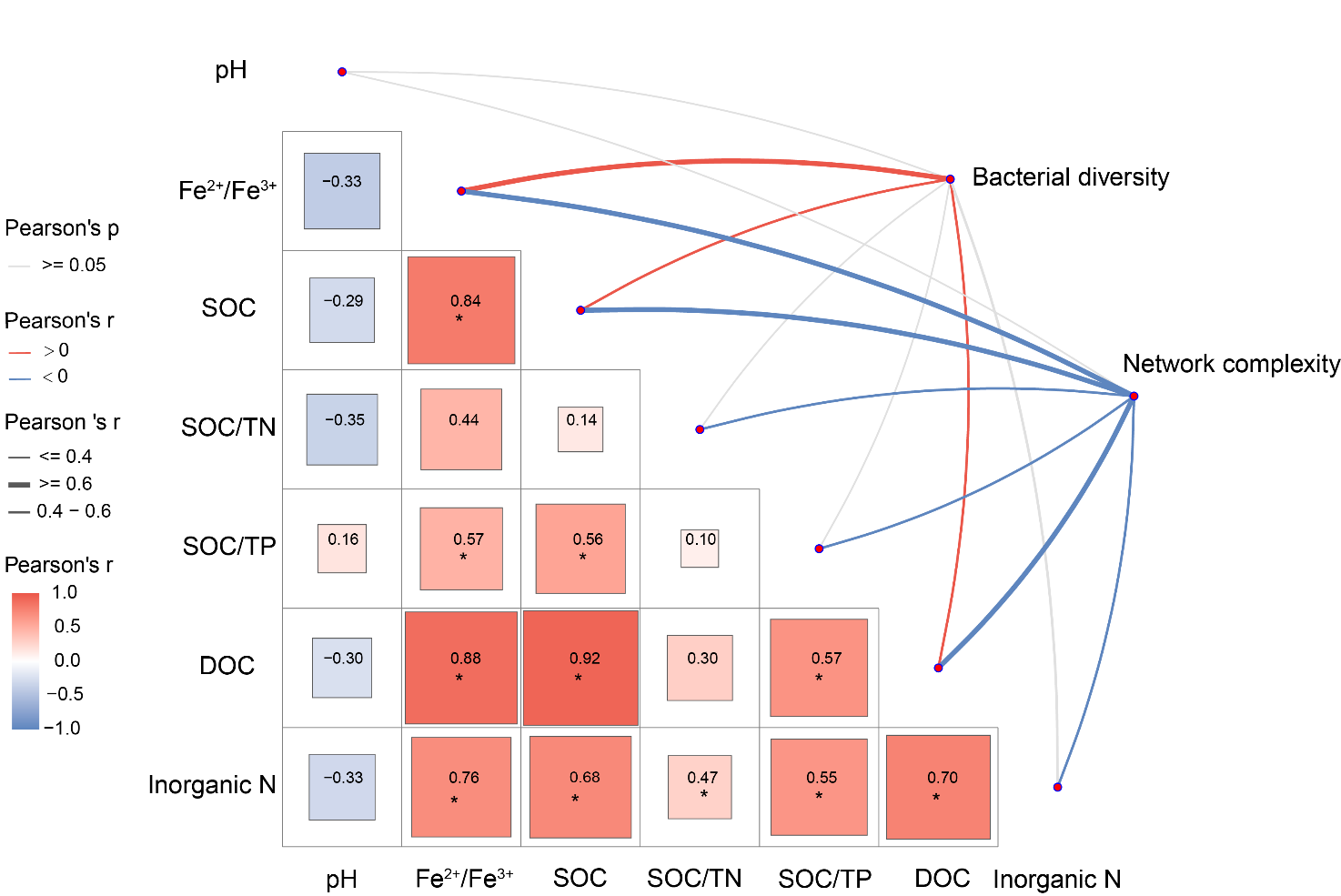
**

**Figure S5.** Random forest analysis shows the relative importance of soil properties on (A) bacterial diversity and (B) network complexity. Bacterial diversity is characterized by the first axis of soil bacterial taxonomic and phylogenetic diversities based on principal component analysis. Taxonomic diversity is characterized by the richness index. Network complexity is characterized by the first axis of topological features of soil bacterial co-occurrence network based on principal component analysis. Blue columns denote significant influences of parameters at *P* < 0.05; grey columns denote insignificant influences of parameters at *P* > 0.05. Fe^2+^/Fe^3+^, the ratio of soil ferrous iron to ferric iron; SOC, soil organic carbon; DOC, dissolved organic carbon; Inorganic N, inorganic nitrogen; SOC/TN, the ratio of soil organic carbon to total nitrogen; SOC/TP, the ratio of soil organic carbon to total phosphorus.


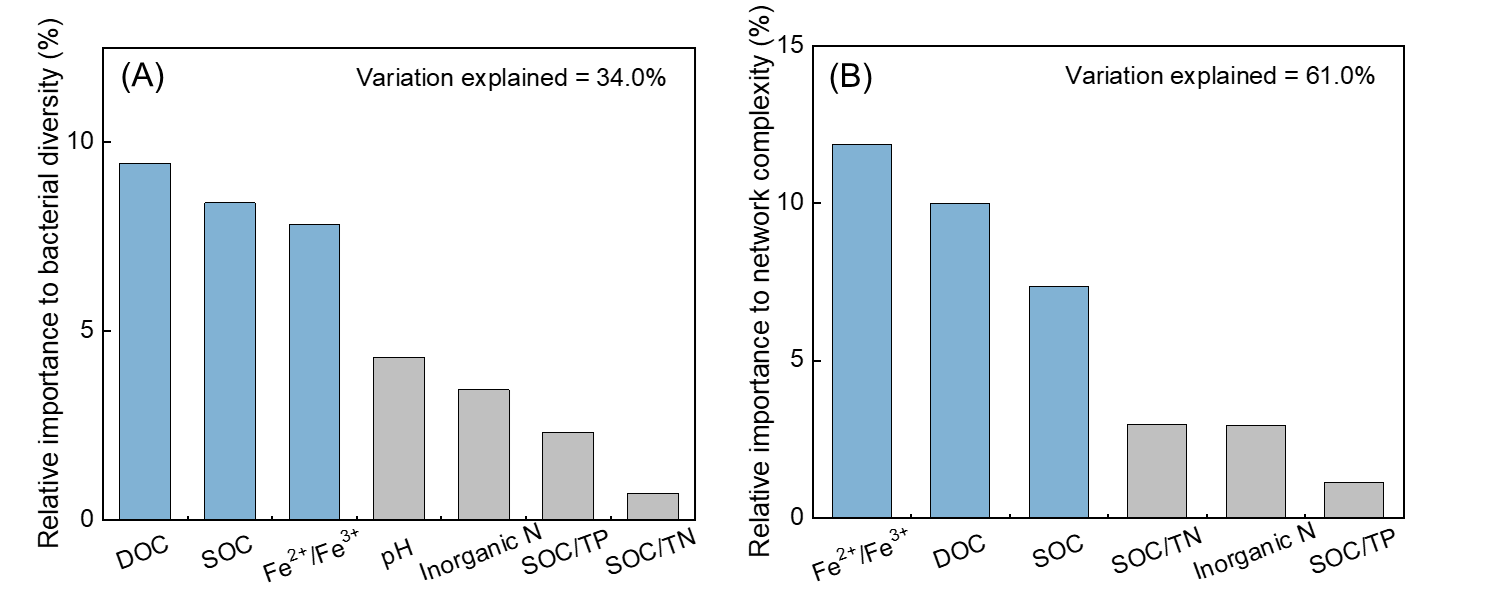


**Figure S6.** Random forest analysis shows the relative importance of soil properties on the richness index of (A) Proteobacteria and (B) Firmicutes. Blue columns denote significant influences of parameters at *P* < 0.05; grey columns denote insignificant influences of parameters at *P* > 0.05. Fe^2+^/Fe^3+^, the ratio of soil ferrous iron to ferric iron; SOC, soil organic carbon; DOC, dissolved organic carbon; Inorganic N, inorganic nitrogen; SOC/TN, the ratio of soil organic carbon to total nitrogen; SOC/TP, the ratio of soil organic carbon to total phosphorus.

**
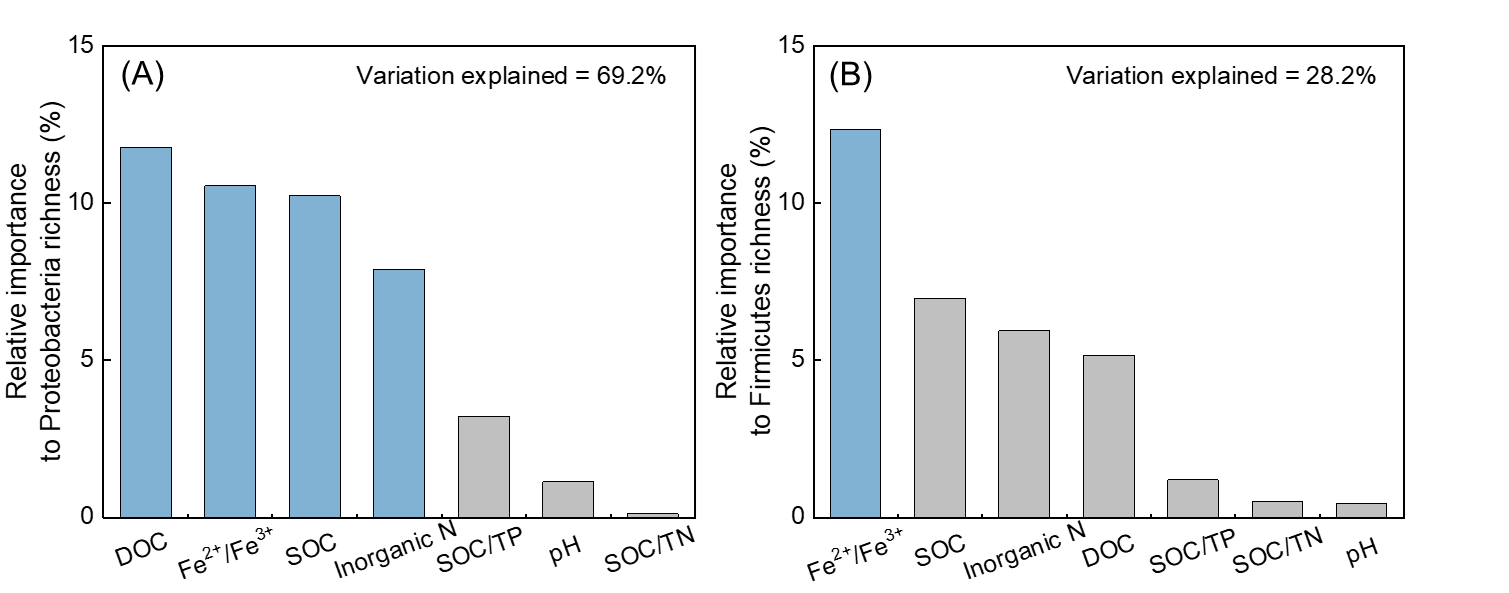
**

**Figure S7.** Linear regression analysis shows the relationships between soil bacterial (A) robustness and (B) vulnerability index and network complexity of soil bacterial communities. Network complexity is characterized by the first axis of topological features of soil bacterial co-occurrence network based on principal component analysis.

**
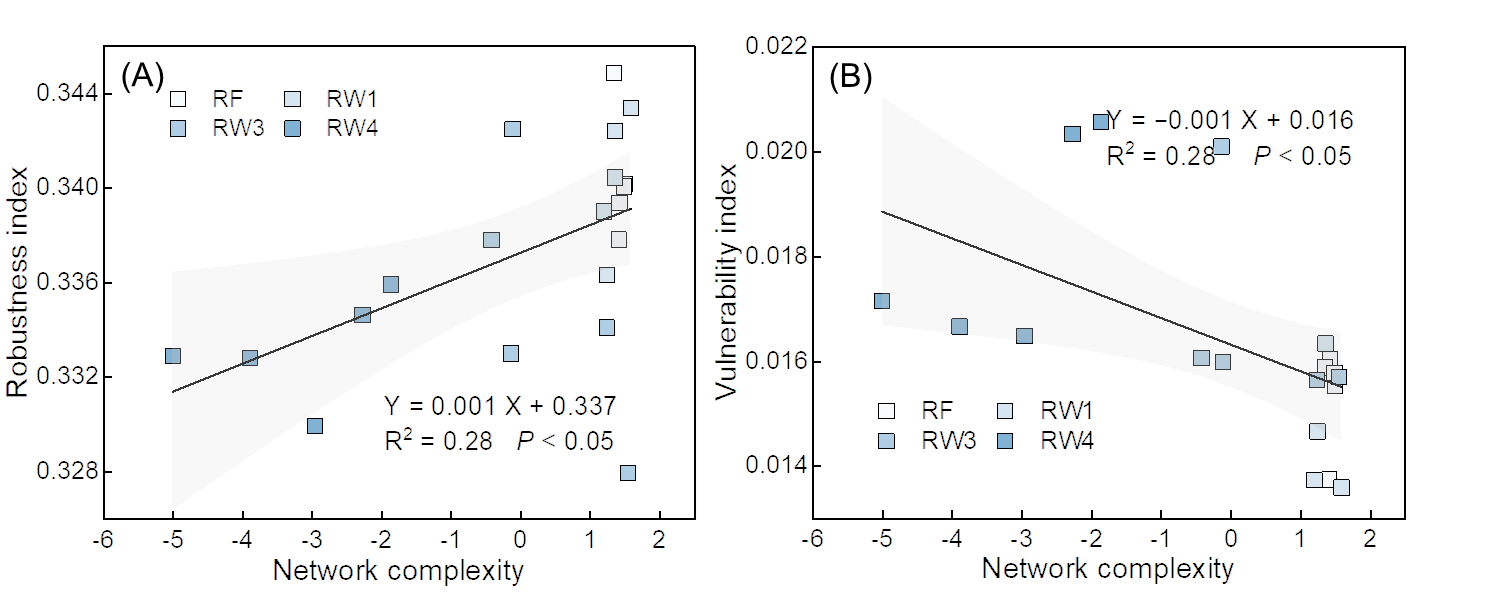
**

**Figure S8.** Assembly processes of soil bacterial communities (A) during wetland restoration after agricultural abandonment and the influence of variations in soil anaerobic condition (B), labile C supply (C) and species association (D) on assembly processes of bacterial communities. In panel A, assembly processes of soil bacterial communities are evaluated by phylogenetic normalized stochasticity ratio (pNST). The boxplots include each box showing mean, median, quartiles, and values within 1.5-times the interquartile range. Different letters indicate significant differences between treatments at *P* < 0.05. In panels B, C and D, variations in soil anaerobic condition, labile C supply and species association are characterized by dissimilarity matrices calculated based on Euclidean distances. Soil anaerobic condition is characterized by soil Fe^2+^/Fe^3+^; soil labile C supply is characterized by DOC content; species association is characterized by topological features of soil bacterial co-occurrence network.

**
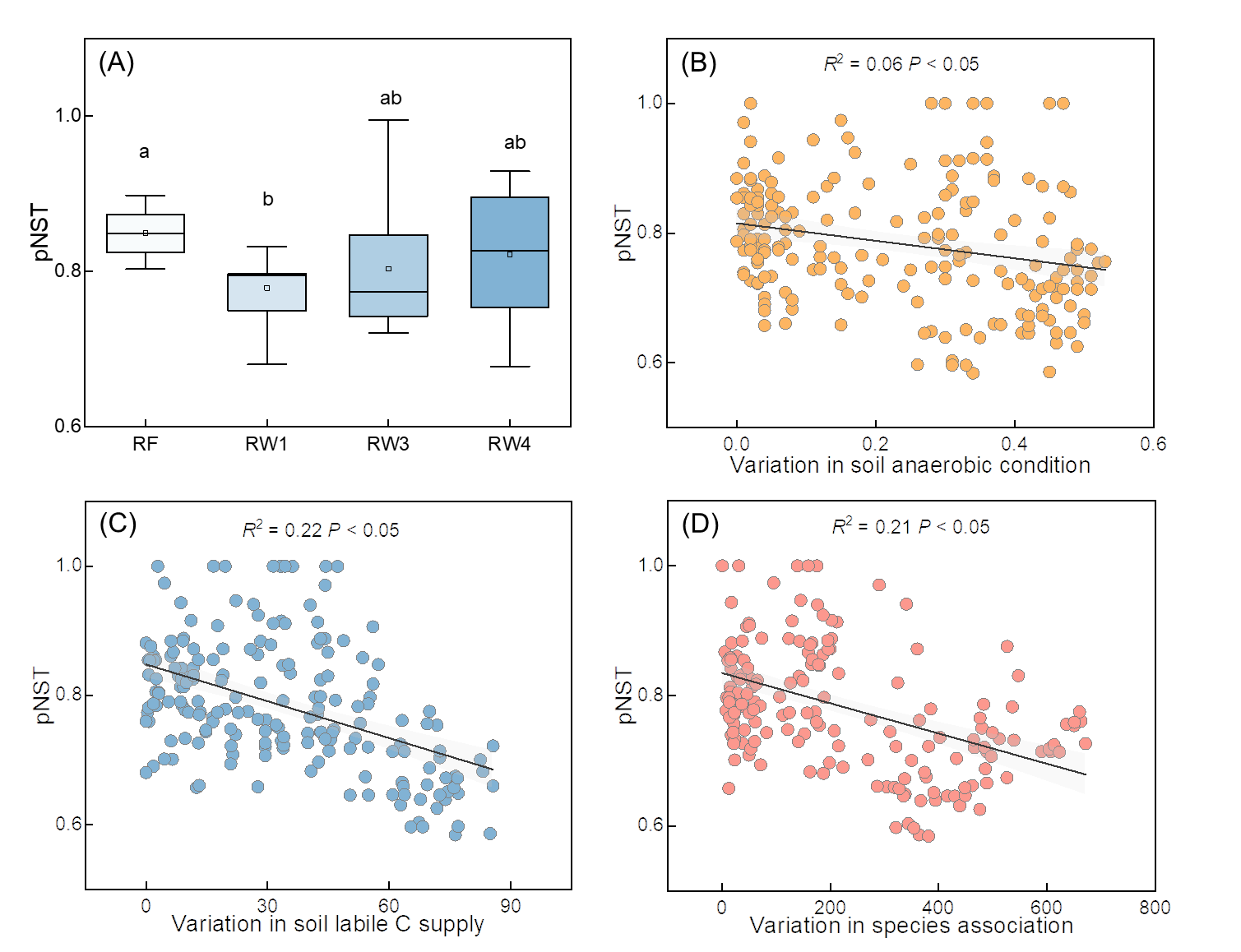
**

**Figure S9.** Linear regression analysis shows the relationships between activities of (A) hydrolase and (B) oxidase and richness index of Bacilli.

**
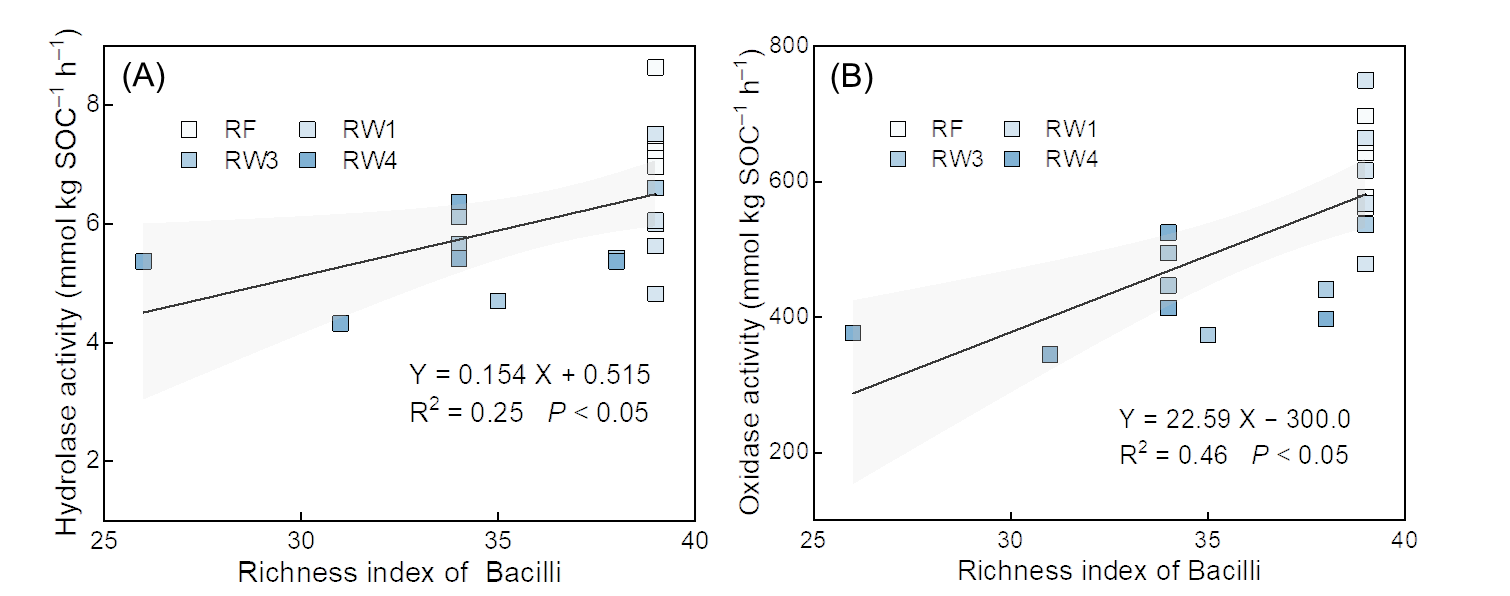
**

**Figure S10.** Effects of wetland restoration after agricultural abandonment on the abundances of hydrolase and oxidase in modules. Vertical bars denote the standard errors of the mean (*n* = 5). Different letters indicate significant differences between treatments at *P* < 0.05.

**
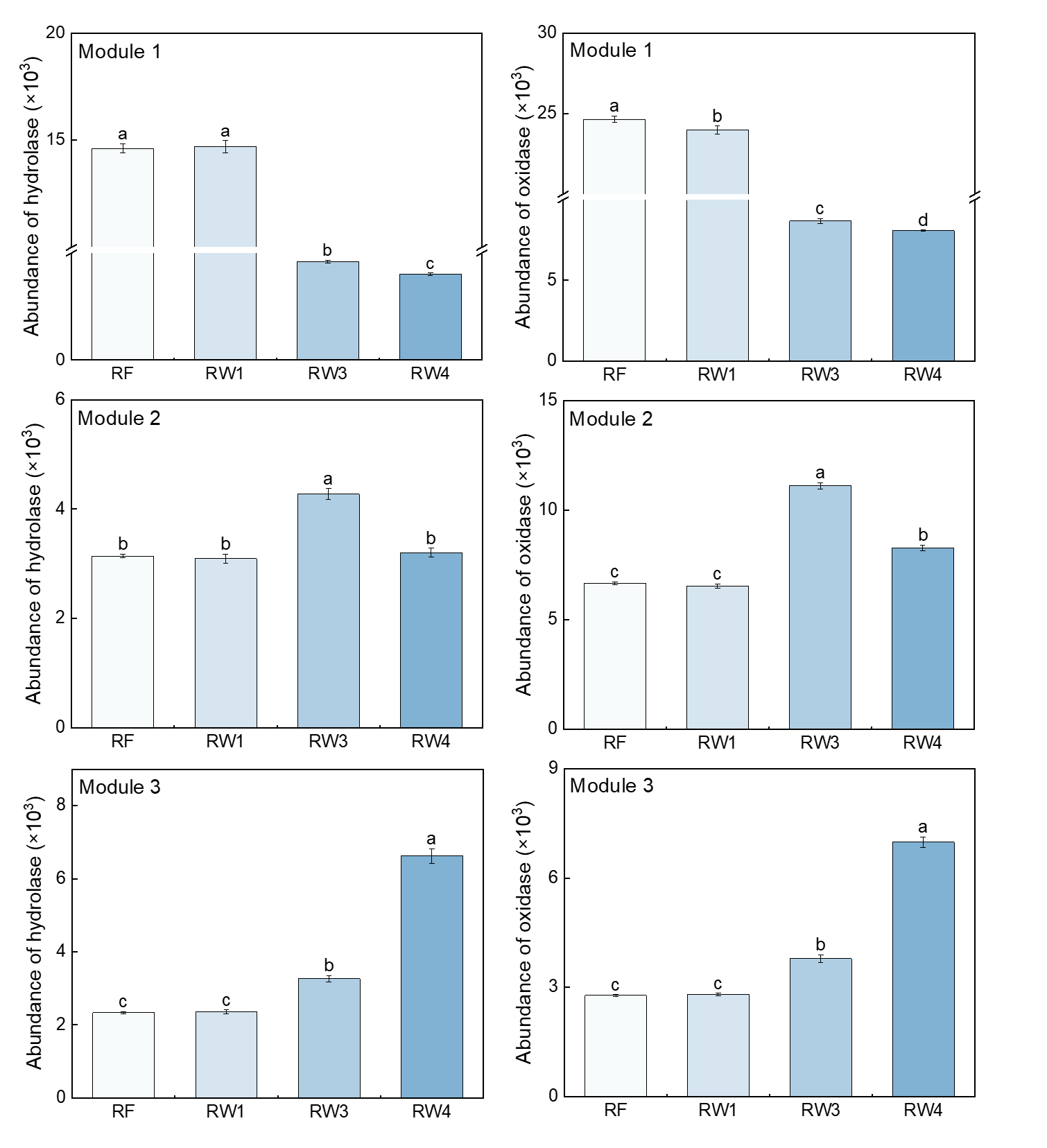
**

**Figure S11.** A priori partial least squares path model based on theoretical knowledge and hypotheses of this study. Cascading effects of soil anaerobic condition, richness index of keystone taxa in keystone module, species association within keystone module, and microbial C metabolism on SOC decomposition.

**
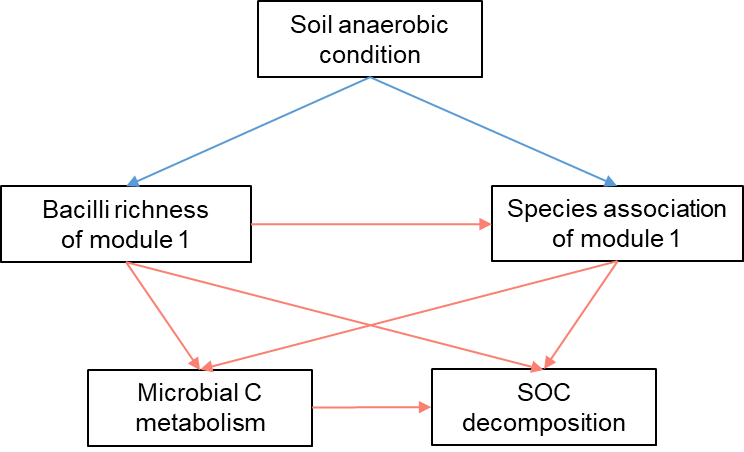
**

**Appendix B. Supplementary** **methods**

**Method 1.** Determination of soil properties

Soil ferrous iron (Fe^2+^) and ferric iron (Fe^3+^) were analyzed using the ferrozine-ultraviolet (UV) absorbance method (Stookey, 1970). Specifically, fresh soil was extracted with 0.5 M hydrochloric acid, and Fe^2+^ was quantified at 562 nm on a UV-visible spectrophotometer (UV-1800; Shimadzu, Kyoto, Japan) after mixing with 5 mM ferrozine solution. Total Fe was first reduced with 2% hydroxylamine hydrochloride and then quantified as Fe^2+^. Fe^3+^ was calculated by subtracting Fe^2+^ from total Fe. Dissolved organic C was extracted with deionized water, followed by centrifugation and filtration (< 0.45 μm), and quantified using the combustion oxidation nondispersive infrared absorption method on a total organic C (TOC) analyzer (Vario TOC Cube; Elementar, Hanau, Germany). Soil inorganic nitrogen content was the sum of ammonium and nitrate. Fresh soil was extracted with 0.5 M potassium sulphate, and the contents of ammonium and nitrate were measured using a colorimetric method on a segmented flow analyzer (San++ System; Skalar Analytical BV, Breda, Netherlands).

**Method 2.** Determination of soil enzyme activities

The activities of hydrolases including β-1,4-glucosidase and cellobiohydrolase were measured using the modified fluorescent-linked substrate microplate method. Specifically, fresh soil was suspended in 50 mM sodium acetate buffer. 200 μL soil slurry along with 50 μL of 200 μM MUB solution, 50 μL of 10 μM MUB solution, and 50 μL buffer solution was dispensed into 96-well microplates served as the sample assays, quench standards, and sample controls, respectively; 200 μL buffer solution followed with 50 μL of 200 μM MUB solution, 50 μL of 10 μM MUB solution, and 50 μL buffer solution was dispensed into 96-well microplates served as the negative control, reference, and blank, respectively. The 96-well microplates were covered and incubated in the dark at 20 °C for 2 h. The reaction was stopped by adding 10 μL of 1 M NaOH, and fluorescence was measured as soon as possible using a microplate fluorometer (Synergy HT; BioTek, Winooski, VT) with 365 nm excitation and 450 nm emission filters.

The activities of oxidases including peroxidase and phenol oxidase were measured using the microplate method. Specifically, fresh soil was suspended in 50 mM sodium acetate buffer. Soil slurry was mixed with 25 μM L-3,4-dihydroxyphenylalanine (DOPA) in the wells of sample assays, and another soil slurry was mixed with buffer solution in the wells of sample controls. Negative wells contained buffer solution and 25 μM L-DOPA, and blank wells only contained buffer solution. Peroxidase activity assay required an additional 0.3% hydrogen peroxide into 96-well microplates. The 96-well microplates were incubated in the dark at 20°C for 5 h for peroxidase and 24 h for phenol oxidase. The activities of oxidases were quantified using a microplate spectrophotometer (Synergy H1; BioTek, Vermont, USA) at 450 nm, and calculated using the following equations:

absorbance = absorbance of (sample assay – sample control – negative control) (1)

activity = (absorbance × 250) / (7.9 × 0.2 × incubation time × soil weight) (2)

where 250 is the volume (mL) of soil slurry; 7.9 is the micromolar extinction coefficient (mmol^–1^); and 0.2 is the sample volume (mL) in the microplate. Incubation time is 5 h for peroxidase and 24 h for phenol oxidase, and soil weight is the oven-dried weight.

**Method 3.** High-throughput sequencing

DNA samples were extracted using a FastDNA Spin Kit for Soil (MP Biomedicals, CA, USA), and concentrations were determined using an ND-1000 ultraviolet-visible spectrophotometer (NanoDrop Technologies, Wilmington, USA). The 16S ribosomal RNA (rRNA) gene was amplified using the universal primer pairs 515F/907R. PCR cycling conditions were stringently followed, starting with initial denaturation at 95°C for 3 min, followed by 35 cycles of denaturation at 95°C for 30 s, annealing at 55°C for 30 s, and extension at 72°C for 45 s. A final extension at 72°C for 10 min was executed to complete the process. PCR reactions were performed in triplicate 20 μL mixture containing 2 μL of 10 × Buffer, 2 μL of 2.5 mM deoxyribonucleotide triphosphate, 0.8 μL of each primer, 0.2 μL of TaKaRa rTaq Polymerase, 0.2 μL of bovine serum albumin and 10 ng of template DNA. The resulting PCR products were extracted from a 2% agarose gel and further purified using the AxyPrep DNA Gel Extraction Kit (Axygen Biosciences, CA, USA). Purified amplicons were pooled in equimolar and paired-end sequenced (2 × 300) on an Illumina MiSeq platform.

Raw fastq files were quality-filtered by Trimmomatic and merged by FLASH with the following criteria: (i) The reads were truncated at any site receiving an average quality score < 20 over a 50 bp sliding window. (ii) Sequences whose overlap was longer than 10 bp were merged according to their overlap with a mismatch of no more than 2 bp. (iii) Sequences of each sample were separated according to barcodes (exactly matching) and Primers (allowing 2 nucleotide mismatching), and reads containing ambiguous bases were removed (Magoč and Salzberg, 2011; Bolger et al., 2014). Operational taxonomic units were clustered with a 97% similarity cutoff using Usearch (Edgar, 2013). The taxonomy of each 16S rRNA gene sequence was annotated using the Silva database (Quast et al., 2012).

**References**

Bolger, A.M., Lohse, M., and Usadel, B. (2014). Trimmomatic: a flexible trimmer for Illumina sequence data. Bioinformatics 30, 2114−2120. doi: 10.1093/bioinformatics/btu170.

Edgar, R.C. (2013). UPARSE: highly accurate OTU sequences from microbial amplicon reads. Nat. Methods 10, 996−998. doi: 10.1038/nmeth.2604.

Magoč, T., and Salzberg, S.L. (2011). FLASH: fast length adjustment of short reads to improve genome assemblies. Bioinformatics 27, 2957−2963. doi: 10.1093/bioinformatics/btr507.

Quast, C., Pruesse, E., Yilmaz, P., Gerken, J., Schweer, T., Yarza, P., et al. (2012). The SILVA ribosomal RNA gene database project: improved data processing and web-based tools. Nucleic Acids Res. 41, D590−D596. doi: 10.1093/nar/gks1219.

Stookey, L.L. (1970). Ferrozine---a new spectrophotometric reagent for iron. Anal. Chem. 42, 779−781. doi: 10.1021/ac60289a016.
